# The transcription factor WRKY41*–FLAVONOID 3′-HYDROXYLASE* module fine-tunes flavonoid metabolism and cold tolerance in potato

**DOI:** 10.1093/plphys/kiaf070

**Published:** 2025-02-20

**Authors:** Huihui Bao, Li Yuan, Yongchao Luo, Jinxiu Zhang, Xi Liu, Qiuju Wu, Xiyao Wang, Jitao Liu, Guangtao Zhu

**Affiliations:** Shenzhen Branch, Guangdong Laboratory of Lingnan Modern Agriculture, Genome Analysis Laboratory of the Ministry of Agriculture and Rural Affairs, Agricultural Genomics Institute at Shenzhen, Chinese Academy of Agricultural Sciences, Shenzhen 518120, China; Yunnan Key Laboratory of Potato Biology, Engineering Research Center of Sustainable Development and Utilization of Biomass Energy, Ministry of Education, School of Energy and Environment Sciences, Yunnan Normal University, Kunming 650500, China; Shenzhen Branch, Guangdong Laboratory of Lingnan Modern Agriculture, Genome Analysis Laboratory of the Ministry of Agriculture and Rural Affairs, Agricultural Genomics Institute at Shenzhen, Chinese Academy of Agricultural Sciences, Shenzhen 518120, China; Yunnan Key Laboratory of Potato Biology, Engineering Research Center of Sustainable Development and Utilization of Biomass Energy, Ministry of Education, School of Energy and Environment Sciences, Yunnan Normal University, Kunming 650500, China; Yunnan Key Laboratory of Potato Biology, Engineering Research Center of Sustainable Development and Utilization of Biomass Energy, Ministry of Education, School of Energy and Environment Sciences, Yunnan Normal University, Kunming 650500, China; Yunnan Key Laboratory of Potato Biology, Engineering Research Center of Sustainable Development and Utilization of Biomass Energy, Ministry of Education, School of Energy and Environment Sciences, Yunnan Normal University, Kunming 650500, China; Yunnan Key Laboratory of Potato Biology, Engineering Research Center of Sustainable Development and Utilization of Biomass Energy, Ministry of Education, School of Energy and Environment Sciences, Yunnan Normal University, Kunming 650500, China; Yunnan Key Laboratory of Potato Biology, Engineering Research Center of Sustainable Development and Utilization of Biomass Energy, Ministry of Education, School of Energy and Environment Sciences, Yunnan Normal University, Kunming 650500, China; Guangdong Provincial Key Laboratory of Crops Genetics and Improvement, Crop Research Institute, Guangdong Academy of Agricultural Sciences, Guangzhou 510640, China; Yunnan Key Laboratory of Potato Biology, Engineering Research Center of Sustainable Development and Utilization of Biomass Energy, Ministry of Education, School of Energy and Environment Sciences, Yunnan Normal University, Kunming 650500, China

## Abstract

Cold stress adversely affects crop growth and productivity. Resolving the genetic basis of freezing tolerance is important for crop improvement. Wild potato (*Solanum commersonii*) exhibits excellent freezing tolerance. However, the genetic factors underlying its freezing tolerance remain poorly understood. Here, we identified *flavonoid 3′-hydroxylase* (*F3′H*), a key gene in the flavonoid biosynthesis pathway, as highly expressed in *S*. *commersonii* compared with cultivated potato (*S. tuberosum* L.). Loss of *ScF3′H* function impaired freezing tolerance in *S*. *commersonii*, while *ScF3′H* overexpression in cultivated potato enhanced its freezing tolerance. Metabolic analysis revealed that F3′H generates more downstream products by adding hydroxyl (−OH) groups to the flavonoid ring structures. These flavonoids enhance reactive oxygen species scavenging, thereby contributing to freezing tolerance. Furthermore, the W-box element in the *F3′H* promoter plays a critical role in cold responses. Cold-induced transcription factor ScWRKY41 directly binds to the *ScF3′H* promoter region and recruits histone acetyltransferase 1 (ScHAC1), which enhances histone acetylation at the *F3′H* locus and activates its transcription. Overall, we identified the cold-responsive WRKY41*–F3′H* module that enhances freezing tolerance by augmenting the antioxidant capacity of flavonoids. This study reveals a valuable natural gene module for breeding enhanced freezing tolerance in potato and other crops.

## Teaser

Under cold conditions, a WRKY transcription factor activates the expression of a flavonoid biosynthesis-related gene, thus enhancing hydroxylated flavonoid levels and freezing tolerance in wild potato.

## Introduction

Cold stress drastically affects plant survival and crop yield globally. As the most important tuber crop worldwide, cultivated potato (*Solanum tuberosum* L.) is highly susceptible to cold, frequently resulting in substantial potato production losses (Pino et al. 2008). Compared with cultivars, several wild potato species exhibit freezing tolerance associated with the expression of cold-responsive genes involved in metabolism, such as flavonoids, sugars, and polyamines (Chen and Li 1980; Oufir et al. 2008; D'Amelia et al. 2018; Kou et al. 2018; Zeng et al. 2019; Liu et al. 2023a, 2023b; Zhu et al. 2023; Feng et al. 2024). *Solanum commersonii* has been identified as the most freezing-tolerant species (Palta et al. 1993), and its genome sequence has recently been released (Feng et al. 2024). *S. commersonii* is an important germplasm resource for the genetic analysis of freezing tolerance in potato breeding.

Flavonoids, a diverse group of plant secondary metabolites, function as reactive oxygen species (ROS) scavengers due to the hydroxyl groups attached to their ring structures and thereby neutralize radicals (Winkel-Shirley 2002; Hernandez et al. 2009). Specifically, anthocyanins, a prominent flavonoid subclass, enhance the cold tolerance of wild potato (Li et al. 2017a, 2017b; D'Amelia et al. 2018). The flavonoid metabolic pathway is initiated by the condensation of malonyl-CoA and 4-coumaroyl-CoA, which is catalyzed by the enzyme chalcone synthase (CHS). This reaction produces chalcone, a flavonoid precursor molecule. Chalcone is then converted to naringenin, a flavanone, through a series of reductions and isomerizations catalyzed by chalcone isomerase (CHI) and flavanone 3-hydroxylase (F3H) (Wen et al. 2020). Flavonoid 3′-hydroxylase (F3′H) and flavonoid 3′,5′-hydroxylase (F3′5′H) introduce hydroxyl groups at the 3′ and 3′,5′ positions of flavonoids, respectively, producing flavonols and anthocyanins with different properties. Another important branch of the flavonoid metabolic pathway involves the conversion of naringenin to anthocyanins, which is catalyzed by anthocyanin synthase (ANS) (Liu et al. 2021). The deficiency of key genes in the flavonoid pathway can disrupt many physiological and biochemical processes, including plant growth, development, and stress responses (Singh et al. 2021; Daryanavard et al. 2023; Gautam et al. 2023).

The flavonoid metabolic pathway is regulated by a number of transcription factors, including MYB, basic helix–loop–helix (bHLH), and WD repeat proteins (Xu et al. 2014; Li et al. 2022). Flavonoid metabolism is also influenced by environmental factors, such as light, temperature, and nutrient availability, allowing plants to adapt to adverse conditions (Li et al. 2017a, 2017b; Ma et al. 2021; Su et al. 2022). In potato, some MYB transcription factors, such as anthocyanin 1 (StAN1), StMYB113, and StMYBA1, have a positive regulatory effect on anthocyanin accumulation in tuber pigmentation, while others, such as StMYB3, StMYB44, and StMYBATV, negatively modulate this process (Liu et al. 2016, 2019; Liu et al. 2023a, 2023b). Recent studies have shown that WRKY transcription factor StWRKY70 promotes anthocyanin accumulation in tubers by interacting with StAN1 (Zhang et al. 2024). However, the role of flavonoid metabolism in regulating the environmental adaptability of potato remains unclear.

In our study, cold-responsive *ScF3′H* was proved to alter the metabolic flow of flavonoids, flavonols, and anthocyanins. The *ScF3′H* knockout mutants exhibited reduced anthocyanin accumulation and increased sensitivity to freezing stress. *ScF3′H* overexpression led to anthocyanin overproduction, which enhanced ROS scavenging and freezing tolerance. Furthermore, the W-box in the *ScF3′H* promoter region was the crucial *cis*-element for cold-induced *ScF3′H* expression. Cold-triggered ScWRKY41 activated *ScF3′H* expression by binding the *cis*-elements and recruiting histone acetyltransferase 1 (ScHAC1). Based on our results, the ScWRKY41-*ScF3′H* module plays a crucial role in modulating anthocyanin biosynthesis and enhancing freezing tolerance.

## Results

### Cold induces *ScF3′H* expression in *S*. *commersonii*

**Figure 1.**
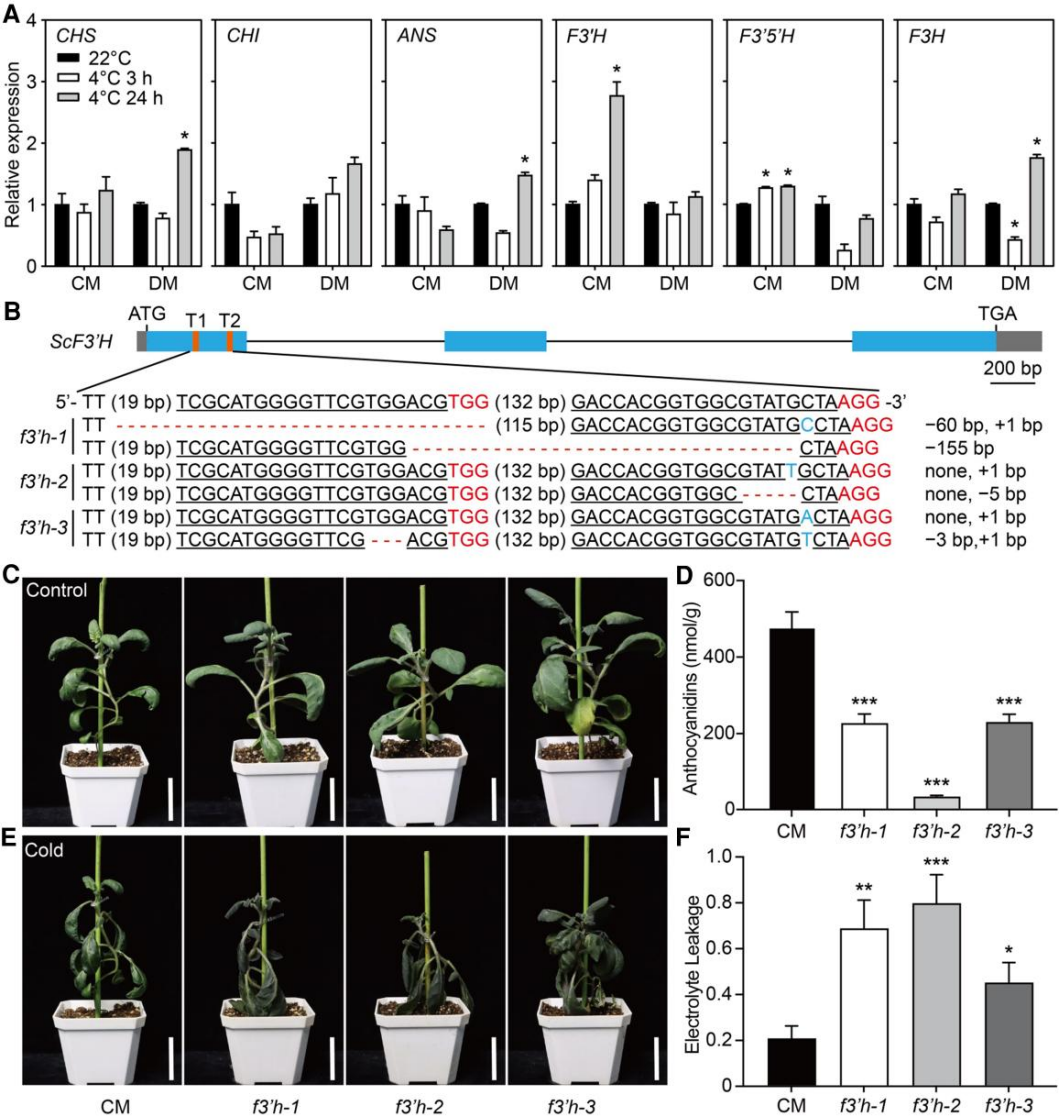
Genome editing of *scf3′h* in *S. commersonii* using the CRISPR/Cas9 system. **A)** RT-qPCR verification of *CHS*, *CHI*, *ANS*, *F3′H*, *F3′5′H*, and *F3H* expression in *S*. *commersonii* (CM) and *S*. *tuberosum* (DM) under cold conditions (4 °C) for 3 and 24 h. *EF1A* was used as the internal reference gene. The data represent the mean ± SE of 3 independent experiments. Asterisks indicate statistically significant differences from 22 °C based on Student's *t-*test (*P* < 0.01). **B)** Targeted mutagenesis of *scf3′h* using CRISPR–Cas9. Protospacer adjacent motif (PAM) sequences are marked as red. The underslining indicates the target site, blue font indicates nucleotide insertions, and dashes indicate base deletions. Three independent mutant lines in one (*f3′h-2*) or two gRNA recognition sites (*f3′h-1* and *f3′h-3*) are shown. **C)** CM and 3 independent transgenic lines of *f3′h* mutants were cultured for 6 wk under normal conditions at 22 °C. **D)** Anthocyanin content (nmol/g) determined in CM and *f3′h* mutant lines. **E)** Plants were transferred to −4 °C for 6 h of treatment. After recovery at 22 °C for 24 h, the plants were photographed and observed for phenotypic changes. **F)** Electrolyte leakage of plants in **(E)** after being subjected to −4 °C treatment for 6 h. The scale bar in **(C)** and **(D)** represents 5 cm. Data in **(E)** and **(F)** represent the mean ± standard error (SE) of 3 independent experiments. Statistical significance was determined by Student's *t*-test (***, *P* < 0.001; **, *P* < 0.01; *, *P* < 0.05).

Flavonoids play important roles in plant tolerance to stress, such as cold, heat, and salt stress (Winkel-Shirley 2002; Hernandez et al. 2009; Li et al. 2017a, 2017b). Anthocyanins, a main class of flavonoids present in wild potato leaves, have been associated with strong cold stress tolerance (D'Amelia et al. 2018). To determine the impact of flavonoid metabolism on the cold tolerance of cold-tolerant genotype CM (*S. commersonii*) and cold-sensitive genotype DM (DM1-3 516R44, *S. tuberosum*), we performed RT-qPCR analysis to assess the expression profiles of key genes in the flavonoid biosynthetic pathway, including *CHS*, *CHI*, *F3′H*, *F3H*, *F3′5′H*, and *ANS*. *CHS*, *CHI*, and *ANS* did not respond to cold in CM but were induced by 4 °C for 24 h in DM. Only the *F3′H* gene showed consistently upregulated expression in CM but showed unchanged expression in DM, indicating the unique function of *F3′H* in CM (Fig. 1A). To characterize the function of *F3′H*, we cloned the gene from CM and named it *ScF3′H*. ScF3′H shared approximately 99% amino acid sequence similarity with that of DM (StF3′H) and was highly conserved in land plants (Supplementary Fig. S1, A and B).

### ScF3′H enhances freezing tolerance in potato

To determine whether *ScF3′H* was responsible for the phenotypic differences in freezing tolerance, we obtained 3 independent knockout lines of the *ScF3′H* gene in CM (*f3′h-1*, *f3′h-2*, and *f3′h-3*) (Fig. 1B). The phenotypes of the *f3′h* mutants and CM were similar during the seedling stage (Fig. 1C). As F3′H is a key enzyme in the anthocyanin biosynthetic pathway, we measured the anthocyanin content of *f3′h* mutants to confirm functional impairment. The anthocyanin content in the *f3′h* mutant was significantly lower than that in CM (Fig. 1D). After freezing treatment (−4 °C for 6 h), *f3′h-1*, *f3′h-2*, and *f3′h-3* exhibited more damage in response to freezing stress (Fig. 1E). Electrolyte leakage was much higher in these mutants than in CM (Fig. 1F). These results indicate that *ScF3′H* has a positive effect on freezing tolerance in potato.

**Figure 2.**
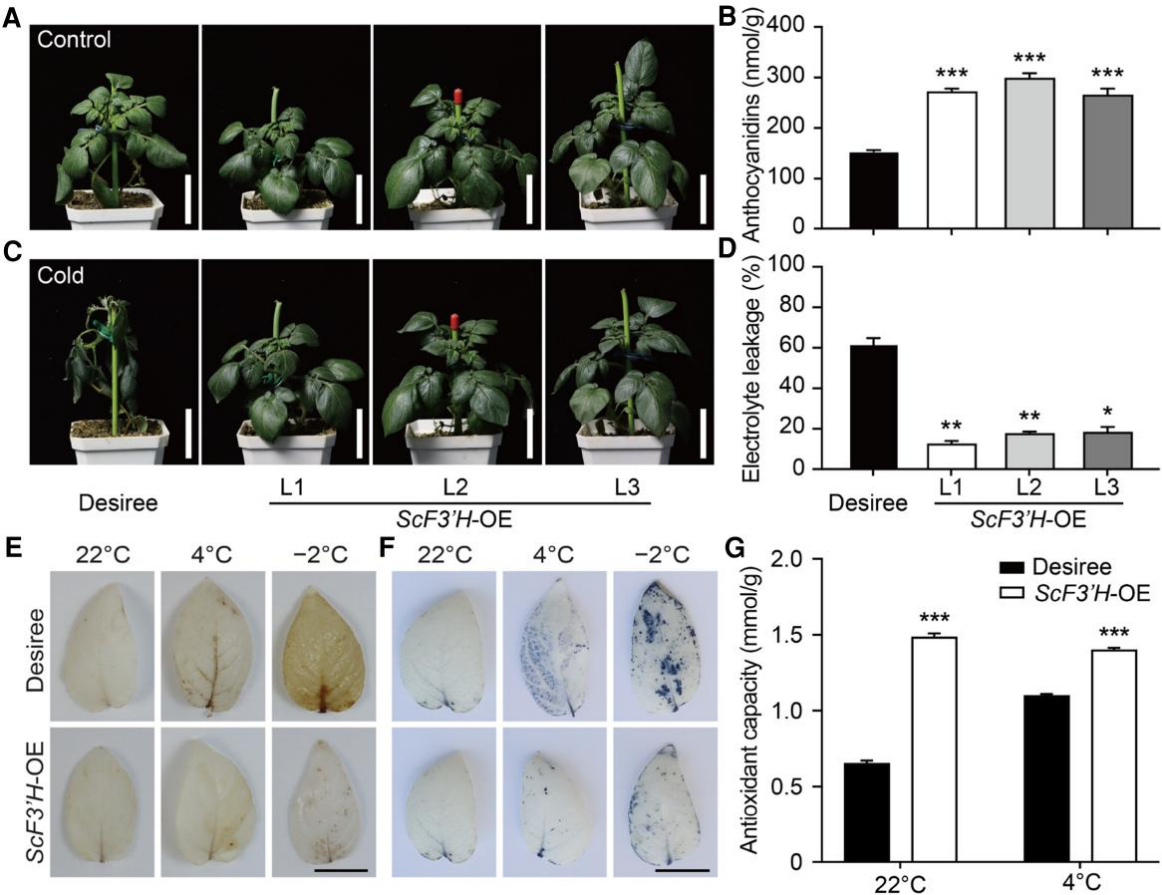
*ScF3′H* OE increases the freezing tolerance of cultivated potatoes. **A)** Desiree and 3 independent transgenic lines (L1, L2, and L3) of *ScF3′H*-OE were cultured for 6 wk under LD conditions at 22 °C. **B)** Anthocyanin content (nmol/g) determined in Desiree and *ScF3′H*-OE lines. **C)** Plants transferred to 4 °C for a 7 d cold acclimation, followed by a 12 h treatment at −2 °C. After recovery at 22 °C for 24 h, the plants were photographed to assess phenotype. **D)** Electrolyte leakage of plants in **(C)** after being subjected to −2 °C treatment for 12 h after cold acclimation. **E** and **F)** DAB **(E)** and NBT **(F)** staining revealed the accumulation of H_2_O_2_ and O^2−^, respectively. The leaves were taken from Desiree and *ScF3′H*-OE grown at 22, 4, and −2 °C. **G)** Antioxidant activity of potato leaf extracts determined using ABTS spectrophotometric methods. The data represent the mean ± SE of 3 independent experiments. The scale bars in **(A)**, **(C)**, and **(E)** represent 5 cm. Statistical significance was determined by Student's *t*-test (***, *P* < 0.001; **, *P* < 0.01; *, *P* < 0.05).

We speculated that low *StF3′H* expression under freezing conditions restricted the freezing tolerance of DM. To verify this hypothesis, an overexpression (OE) assay of *ScF3′H* was conducted in *S*. *tuberosum* L. “Desiree” (Fig. 2A). Three independent OE lines (L1, L2, and L3) were obtained (Supplementary Fig. S2A). RT-qPCR showed that the target gene was highly expressed in these lines (Supplementary Fig. S2B). The total anthocyanidin assay showed that all OE lines accumulated a higher anthocyanidin content than Desiree (Fig. 2B). These OE lines showed enhanced freezing tolerance compared with Desiree, with much lower electrolyte leakage (Fig. 2, C and D). As a significant class of antioxidants, anthocyanins play a crucial role in maintaining the balance of ROS during stress responses (Mittler et al. 2022). To assess whether differentially accumulated anthocyanins in Desiree and OE lines affected the ROS scavenging capacity, two independent experiments were conducted. Desiree leaves stained with DAB (Fig. 2E) and NBT (Fig. 2F) exhibited significantly darker hues after treatment at 4 and −2 °C, indicating substantial accumulation of hydrogen peroxide (H_2_O_2_) and superoxide anions (O^2−^), respectively. In contrast, the *ScF3′H*-OE leaves displayed limited brown and blue coloration, suggesting lower ROS accumulation. An ABTS assay was also used to quantify the antioxidant capacity of the extracts from *ScF3′H*-OE leaves. At 22 °C, the antioxidant capacity of *ScF3′H*-OE leaves exceeded that of Desiree (Fig. 2G). Moreover, at 4 °C, the antioxidant capacity of *ScF3′H*-OE was significantly higher than that of Desiree. These results indicate that *ScF3′H* enhances freezing tolerance by increasing the ROS scavenging capability.

### ScF3′H modulates the metabolic flux of flavonoids

**Figure 3.**
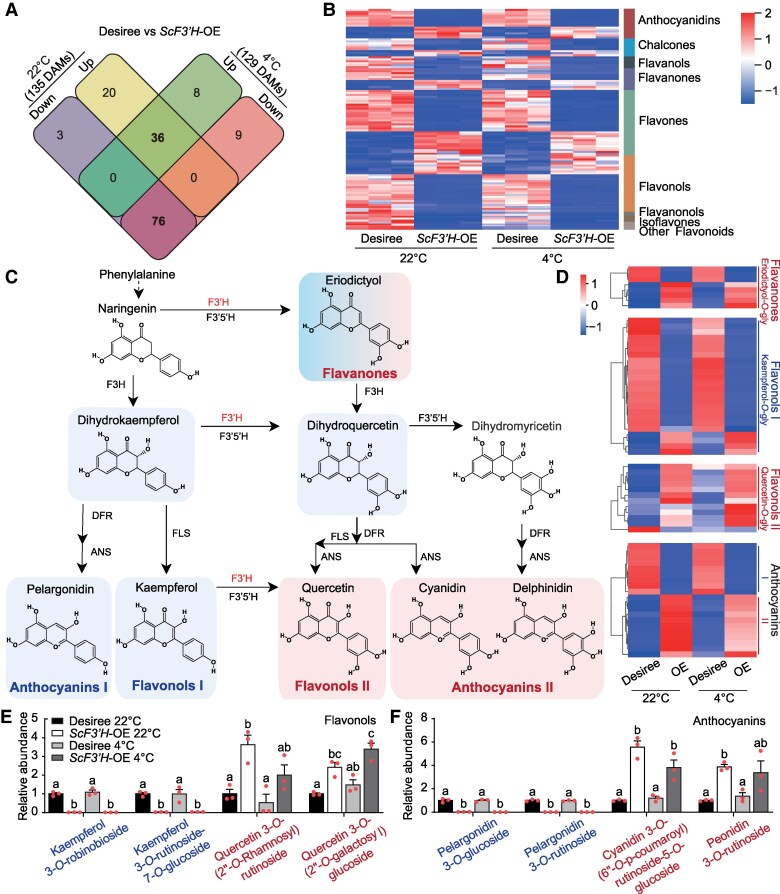
*ScF3′H*-OE enhances the synthesis of quercetin-*O*-glycoside and downstream anthocyanins. **A)** Venn diagram showing the DAMs of flavonoids between Desiree and *ScF3′H*-OE lines grown at 22 and 4 °C. **B)** Heatmap of 112 shared DAMs between Desiree and *ScF3′H*-OE at 22 and 4 °C. The red-to-blue color gradient in **(B)** and **(D)** represents the *z-score* normalized flavonoid metabolomic profile, with red denoting elevated abundance levels and blue signifying reduced abundance levels. **C)** Simplified schematic of the potato flavonoid pathway showing the locations of the enzymatic steps altered in *ScF3′H*-OE lines. Enzyme names are abbreviated as follows: F3H, flavanone 3-hydroxylase; F3′H, flavonoid 3′-hydroxylase; F3′5′H, flavonoid 3′,5′-hydroxylase; FLS, flavonol synthase; DFR, dihydroflavonol 4-reductase; ANS, anthocyanidin synthase. The F3′H enzyme that are the focus of this study are highlighted in red. Boxed text identifies the 5 major flavonoid end products in potato. Blue areas in **(C, D**, **E, F)** indicate downregulation of kaempferol derivatives, and red areas indicate upregulation of quercetin derivatives in *ScF3′H*-OE lines. **D)** Heatmap showing eriodictyol-*O*-gly (Flavanones), kaempferol-*O*-gly (Flavonols I), quercetin-*O*-gly (Flavonols II), and anthocyanins (I and II). **E** and **F)** Changes in flavonols **(E)** and anthocyanins **(F)** between Desiree and *ScF3′H*-OE lines at 22 and 4 °C. The data represent the mean ± SE (*n* = 3). Different letters indicate statistically significant differences among samples (*P* < 0.05, one-way ANOVA with Tukey's multiple comparisons test).

Given the pivotal role of F3′H in flavonoid biosynthesis, we undertook a metabolomic analysis to elucidate the flavonoid metabolic reprogramming triggered by *ScF3′H* OE. Under 22 °C conditions, *ScF3′H*-OE led to alterations in the accumulation of 135 flavonoids, with 56 increased and 79 decreased levels. Under 4 °C conditions, 129 flavonoids were altered in *ScF3′H*-OE lines, 44 flavonoids were upregulated, and 85 flavonoids were downregulated. Collectively, 56% (36) and 86% (76) of shared differentially accumulated metabolites (DAMs) exhibited increased and decreased levels in these two conditions, respectively (Fig. 3A and Supplementary Fig. S3 and Supplementary Tables S1 to S3). This demonstrated that *ScF3′H* OE significantly affected flavonoid metabolic flux. The levels of flavanonols, isoflavones, flavanols, and other flavonoids among the DAMs were uniformly reduced, whereas those of chalcones, flavanones, flavones, flavonols, and anthocyanidins exhibited an inconsistent response, being either upregulated or downregulated (Fig. 3B).

Flavonols and anthocyanins have been reported to play important roles in the cold stress response (Winkel-Shirley 2002). F3′H is a key enzyme regulating anthocyanin and flavonol synthesis. Therefore, we focused on the metabolites that were consumed or generated by the ScF3′H enzymatic reaction. Dihydrokaempferol, kaempferol-*O*-glys, and pelargonidin accumulation were significantly reduced, while the accumulation of eriodictyol-*O*-glys, quercetin-*O*-glys, and related anthocyanins was induced in *ScF3′H*-OE materials (Fig. 3, C and D). Among these, quercetin has been shown to exhibit enhanced radical scavenging activity, which is attributed to the presence of a greater number of hydroxyl (−OH) groups on its aromatic B ring^35^. Furthermore, the changes in flavonoid metabolism in *ScF3′H*-OE at 4 °C were strikingly similar to those observed at 22 °C (Fig. 3, A and B). Specifically, kaempferol-*O*-glys and pelargonidin accumulation were significantly reduced in *ScF3′H*-OE, with no discernible differences between the 22 and 4 °C conditions (Fig. 3, E and F). Similarly, the products of F3′H, flavonols and anthocyanins exhibited consistent and significant upregulation at both 22 and 4 °C (Fig. 3, E and F). Furthermore, these metabolites were nearly unaffected by cold treatment in Desiree and the OE lines, indicating that cold-induced *ScF3′H* transcription is a key contributor to the enhanced freezing tolerance of CM. In general, our results showed that ScF3′H conferred freezing tolerance to potato by accumulating flavonoids with a higher degree of hydroxylation on the B-ring.

### ScWRKY41 binds to the W-box region of the ScF3′H promoter

**Figure 4.**
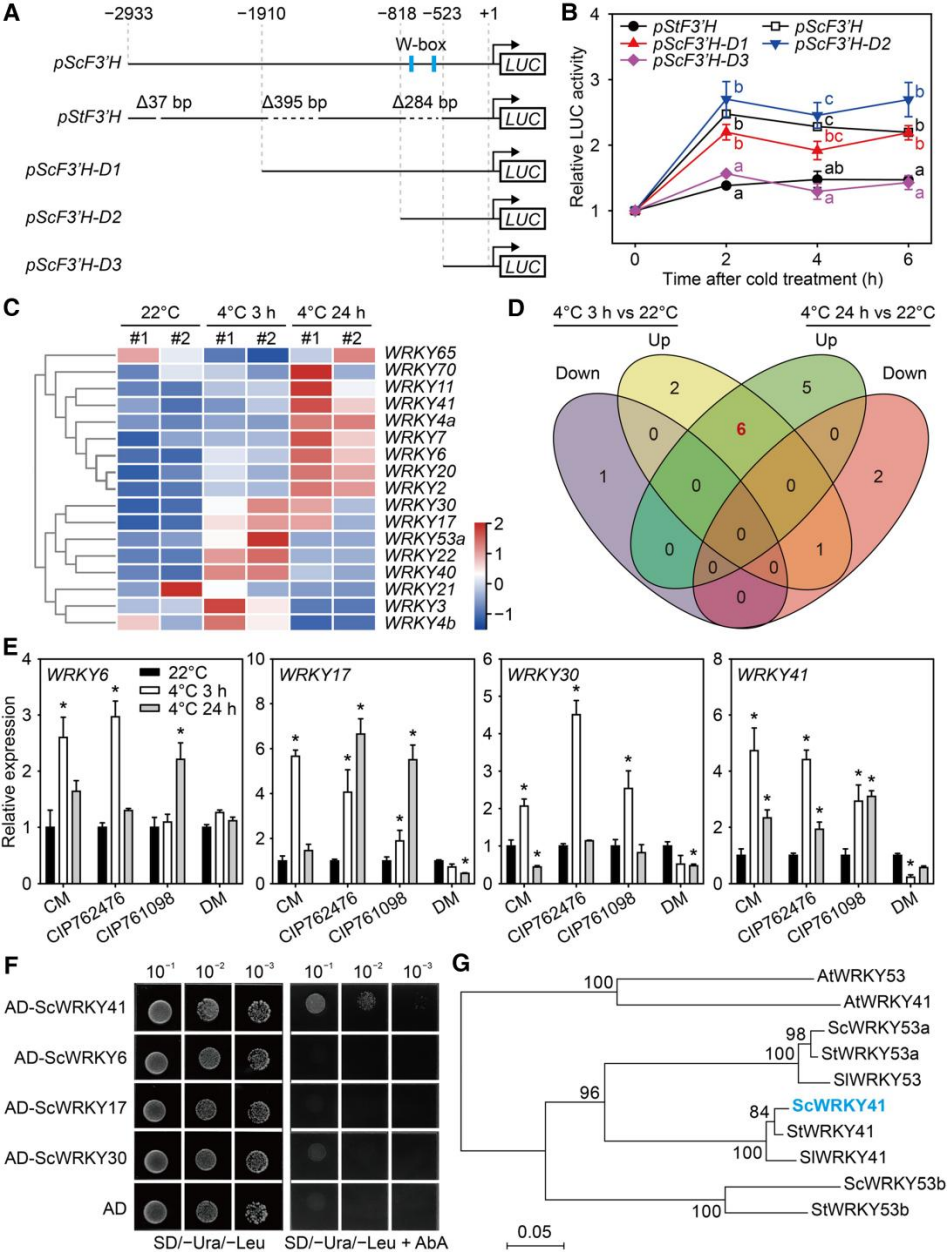
Cold-induced ScWRKY41 binds to the *ScF3′H* promoter. **A)** Schematic representation of the *F3′H* promoter in DM (*pStF3′H*) and CM (*pScF3′H*) and the truncated promoter of *ScF3′H* (*pScF3′H*-*D1*, *pScF3′H*-*D2*, and *pScF3′H*-*D3*). **B)** Response of promoter activity to cold stress. *Agrobacterium rhizogene* MSU440 harboring the *promoter:LUC* constructs shown in **(A)** was used to infect CM. Transgenic roots were cultured at 22 °C for 2 wk and then treated at 4 °C. Reporter gene activity was assessed by measuring luminescence in hairy roots with the luminescence at 0 h normalized to 1. Each value represents the mean ± SE of 5 independent experiments. Different letters indicate statistically significant differences among samples (*P* < 0.05, one-way ANOVA with Tukey's multiple comparisons test). **C)** Heatmap of 17 *WRKYs* in CM from RNA-seq (FPKM > 1; FoldChange > 2). The red-to-blue color gradient represents the normalized RNA-seq data, with red denoting elevated abundance levels and blue signifying reduced abundance levels. **D)** Venn diagram showing the *WRKYs* in CM under control (22 °C) and cold stress (4 °C, 3 and 24 h) conditions. The number “6” represents *WRKYs* that were upregulated under cold stress for both 3 and 24 h compared with the 22 °C conditions. **E)** Expression of candidate *WRKY* genes was verified by RT-qPCR. All materials were grown at 22 °C for 3 wk and transferred to 4 °C. Samples were collected before cold treatment and 3 and 24 h after treatment, using *EF1A* as the internal reference gene. The data represent the mean ± SE of 3 independent experiments. Asterisks indicate statistically significant differences from 22 °C based on Student's *t-*test (*P* < 0.01). **F)** Yeast one-hybrid assay results showing the binding of *WRKYs* to the W-box in the *ScF3′H* promoter. The transformants were grown on synthetic dropout medium without uracil (Ura) and leucine (Leu) or without Ura, Leu, and 200 ng/mL aureobasidin (AbA) to observe their growth phenotypes. **G)** Phylogenetic analysis was performed on the WRKY41 sequences. The neighbor-joining phylogenetic tree of WRKY41 homologous proteins from different plant species was constructed using MEGA 7.0. Bootstrap values were based on 1,000 replicates. The 0.05 scales show substitution distance.

To elucidate the cold-responsive transcriptional regulation of *ScF3′H*, we cloned the promoter sequences of *F3′H* in CM (*pScF3′H*) and DM (*pStF3′H*). In the cold-sensitive DM, 3 large deletions were identified (Fig. 4A). To identify the critical variations of *pScF3′H*, 3 *pScF3′H* constructs with varying lengths were generated to drive *luciferase* (*LUC*) expression (Fig. 4A). A luciferase reporter assay was conducted after hairy root transformation in potato. Two hours of cold treatment resulted in a nearly 3-fold increase in *pScF3′H* activity, while no significant response was observed for *pStF3′H*. Furthermore, the activities of *pScF3′H-D1* and *pScF3′H-D2* were markedly enhanced by cold, whereas *pScF3′H-D3*, similar to *pStF3′H*, exhibited no response to cold induction (Fig. 4B). These findings indicate that the 284 bp region in *pScF3′H* is pivotal for the cold stress response.

Further analysis of the insertion sequence (284 bp) in CM identified two W-box *cis*-elements, which were the potential binding sites of WRKY transcription factors. We obtained 17 cold-responsive *WRKYs* from the RNA-seq data and used them to identify the possible upstream regulons (Fig. 4C and Supplementary Table S4). Among them, 6 *WRKYs* (namely *WRKY3*, *WRKY11*, *WRKY17*, *WRKY30*, *WRKY40*, and *WRKY41* according to their homologies in Arabidopsis) were upregulated at both 3 and 24 h under cold stress (Fig. 4D). WRKY11 and WRKY40 have been reported as transcriptional suppressors in Arabidopsis (Journot-Catalino et al. 2006; Chen et al. 2010). Thus, the remaining 4 WRKYs were validated as candidate transcription factors. Three freezing-resistant *S. commersonii* genotypes and the freezing-sensitive DM genotype were selected for RT-qPCR verification at 4 °C for 3 and 24 h. The expression of *WRKY6*, *17*, *30*, and *41* was upregulated in *S*. *commersonii* genotypes but was downregulated or remained unchanged in DM following cold stress (Fig. 4E). We performed a yeast one-hybrid (Y1H) experiment using the 284-bp region of *pScF3′H*, and found that ScWRKY41 bound to this region, while ScWRKY30 exhibited weak binding potential (Fig. 4F). Consequently, our scrutiny has been exclusively concentrated on ScWRKY41. The potato genome contains 3 homologs of WRKY41, namely StWRKY41, StWRKY53a, and StWRKY53b. The protein sequences of ScWRKY41 and StWRKY41 were 98% similar (Fig. 4G). These results demonstrated that ScWRKY41 could bind to the W-box region of the *ScF3′H* promoter.

### ScWRKY41 activates ScF3′H expression by recruiting ScHAC1

**Figure 5.**
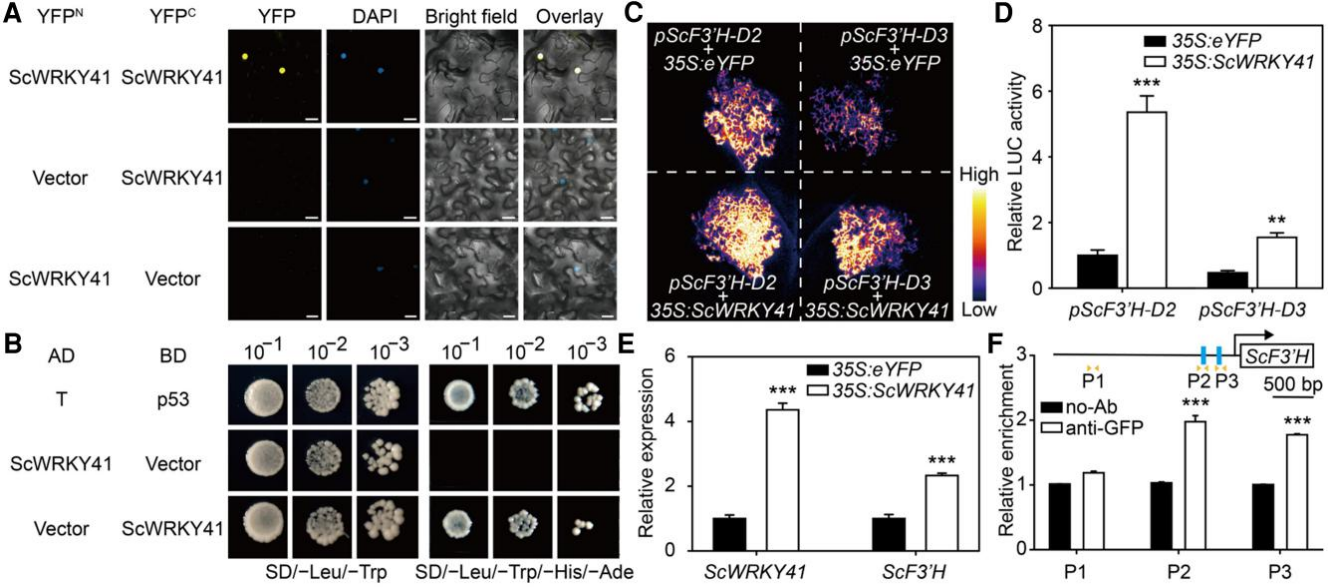
ScWRKY41 activates *ScF3′H* expression. **A)** BiFC assays for the physical self-interaction of ScWRKY41 *in planta*. The N- or C-terminus of YFP was fused with full-length ScWRKY41, with empty vectors serving as negative controls. The scale bar indicates 20 *μ*m. **B)** Y2H assays demonstrated the activation activity of ScWRKY41 in yeast cells. Full-length ScWRKY41 was fused with the DNA-binding domain of GAL4 (BD) and the activation domain of GAL4 (AD) and co-transformed with empty vectors into yeast cells. **C** and **D)** Regulation of *ScWRKY41* on *ScF3′H* promoter activity in *N. benthamiana* leaves. The *pScF3′H-D2* and *pScF3′H-D3* reporter constructs were co-transfected separately with *35S:eYFP* and *35S:ScWRKY41-eYFP* into different regions of *N. benthamiana* leaves, and 48 h after infection, bioluminescence signals were observed using a bioluminescence imaging system **(C)** and an enzyme marker detector **(D)**. **E)** Expression analysis of *ScWRKY41* and *ScF3′H* in the CM hairy roots of *35S: eYFP* and *35S:ScWRKY41-eYFP* by RT-qPCR. **F)** Analyzing the binding of ScWRKY41 to the promoter region of *ScF3′H* using ChIP-qPCR. In the schematic of the *ScF3′H* promoter, the rectangles represent the W-box *cis*-elements, and the triangle arrows represent the position of the primers. ChIP-qPCR assay of fragments containing the W-box *cis*-elements in the promoter regions of *ScF3′H* using Anti-GFP to precipitate *ScWRKY41-eYFP* expressed in the positive hairy roots of *35S:ScWRKY41-eYFP*. A sample without antibody (no-Ab) was utilized as the negative control. Values in **(D)**, **(E)**, and **(F)** are means ± SE from 3 independent experiments, and statistical significance was determined by Student's *t*-test (***, *P* < 0.001; **, *P* < 0.01).

In Arabidopsis, AtWRKY41 and AtWRKY53 belong to the group III WRKY subfamily, and AtWRKY53 has been reported to regulate downstream genes by forming dimers with AtWRKY18 (Ding et al. 2014; Chen et al. 2019). ScWRKY41 has the nuclear localization signal peptide (Supplementary Fig. S4A, red line) and conserved domain of the group III WRKY subfamily (Supplementary Fig. S4A, blue box). Subcellular localization showed that ScWRKY41 was localized in the nucleus and formed homologous dimers (Fig. 5A and Supplementary Fig. S4B). ScWRKY41 showed transcriptional activation activity in yeast cells (Fig. 5B). Using a *Nicotiana benthamiana* leaf-based transient expression system, we investigated the regulatory effect of ScWRKY41 on *pScF3′H* activity. The co-transfection of *pScF3′H-D2* and *pScF3′H-D3* with *35S:ScWRKY41* into *N. benthamiana* leaves resulted in significant enhancement of *pScF3′H-D2* activity. In contrast, ScWRKY41 exhibited a weak regulatory effect on *pScF3′H-D3* (Fig. 5, C and D). We employed a hairy root system to overexpress *ScWRKY41* in CM, using an empty vector as the control. RT-qPCR analysis was performed following the establishment of hairy roots. The expression of the *ScF3′H* gene was significantly elevated compared with the control following *ScWRKY41* OE (Fig. 5E). To evaluate the in vivo binding of ScWRKY41 to the *ScF3′H* promoter region, we conducted ChIP-qPCR analysis using CM hairy roots of *35S:ScWRKY41-eYFP*. The ChIP-qPCR results demonstrated that ScWRKY41 specifically binds to the W-box *cis*-element region within the *ScF3′H* promoter (Fig. 5F). These results indicate that ScWRKY41 activates *pScF3′H* activity and that the W-box elements in its promoter region are essential for this activation.

**Figure 6.**
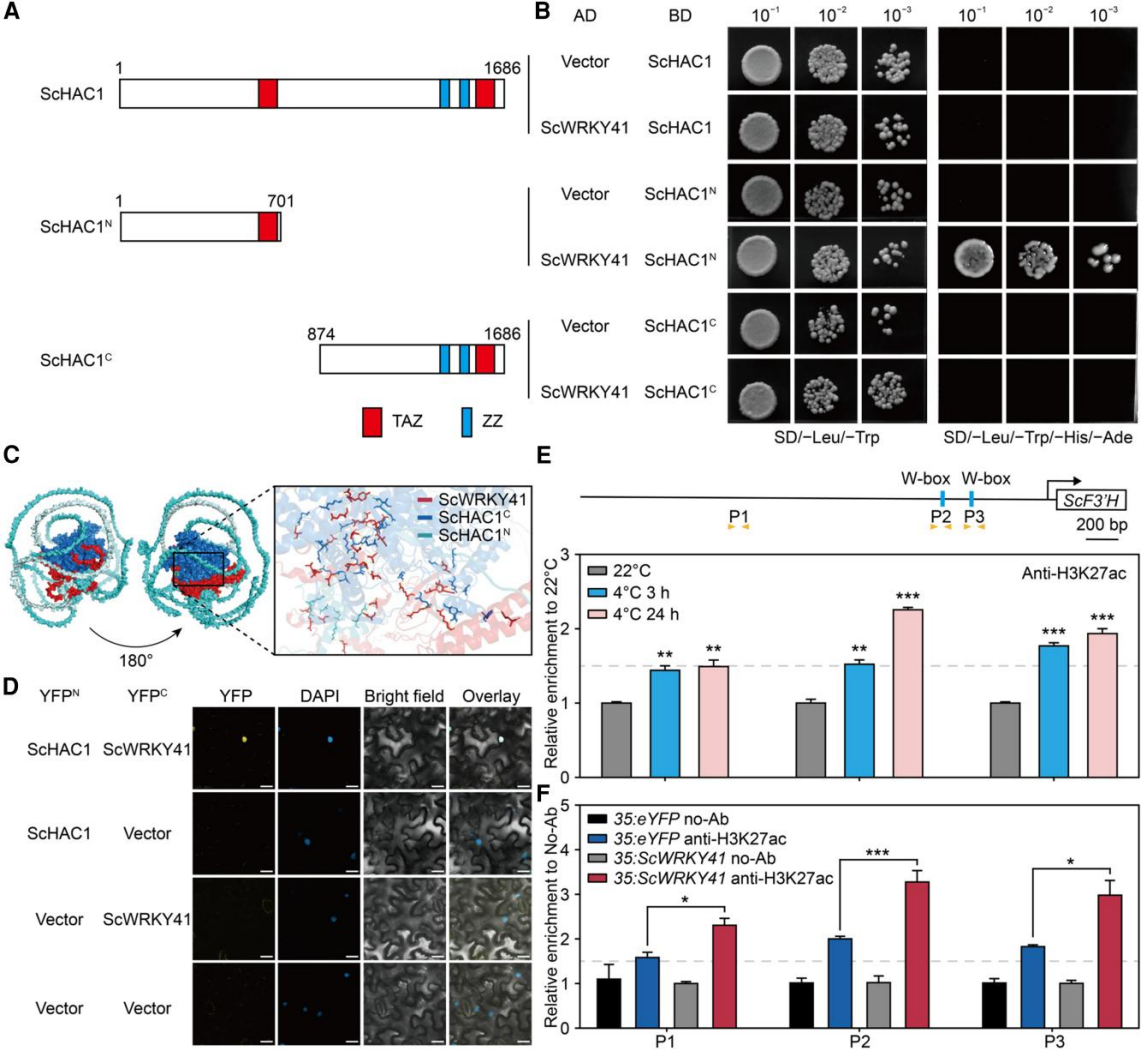
ScWRKY41 recruits ScHAC1 to enhance H3K27ac levels in the *ScF3′H* promoter. **A)** The position of various fragments of HAC1 used for yeast two-hybrid assays (Y2H). TAZ: transcription adaptor putative zinc finger domain; ZZ: Zinc DNA-binding domains; numbers indicate amino acid position. **B)** Y2H assays demonstrated the interaction between HAC1 fragments (as shown in A) and WRKY41. The full-length N- or C-terminus of HAC1 was fused with the DNA-binding domain of GAL4 (BD); full-length ScWRKY41 was fused with the activation domain of GAL4 (AD). **C)** Analysis of the 3-dimensional structures of the ScWRKY41 and ScHAC1 proteins. **D)** BiFC assays were conducted to investigate the physical interaction between ScHAC1 and ScWRKY41 *in planta*. The scale bar indicates 20 *μ*m. **E** and **F)** Analysis of the H3K27ac level in the *ScF3′H* promoter region using ChIP-qPCR. The assay was performed on fragments containing the putative W-box *cis*-elements in the *ScF3′H* promoter regions, using Anti-H3K27ac to precipitate the H3K27ac proteins from CM treated with cold stress for 3 and 24 h **(E)**, as well as from CM hairy roots elicited by *35S:ScWRKY41*  **(F)**. The promoter region of P1 was used as the negative control. In the schematic of the *ScF3′H* promoter, the rectangles represent the W-box *cis*-elements, and the triangle arrows represent the position of the primers. Values in **(E)** and **(F)** are the mean ± SE from 3 independent experiments, and statistical significance was determined by Student's *t*-test (***, *P* < 0.001; **, *P* < 0.01).

In Arabidopsis, AtWRKY18 and AtWRKY53 form a heterodimer that activates the target gene via interaction with AtHAC1, which facilitates the acetylation of lysine 27 on histone H3 (H3K27ac) (Chen et al. 2019). Using a yeast two-hybrid (Y2H) assay, we found that ScWRKY41 interacted with the N-terminus of ScHAC1 (Fig. 6, A and B). Utilizing the docking feature of AlphaFold3 (Abramson et al. 2024), we simulated the 3D structure of the ScWRKY41-ScHAC1 complex and discerned substantial affinity regions at both the N-terminal and C-terminal of ScWRKY41 (Fig. 6C). Distinct YFP fluorescent signals were observed in the nuclei of epidermal cells following co-transformation of ScWRKY41 fused to the C-terminal half of YFP (cYFP) with the full-length ScHAC1 fused to the N-terminal half of YFP (nYFP) in *Nicotiana benthamiana* leaves. BiFC assays were employed to confirm the interaction between ScWRKY41 and ScHAC1 (Fig. 6D). The level of H3K27ac in the *ScF3′H* promoter region gradually increased with prolonged cold treatment, mirroring the pattern of *ScF3′H* transcriptional response to cold stress (Fig. 1A and Fig. 6E). H3K27 acetylation increases gene expression by enhancing the accessibility of DNA inside chromatin (Lin et al. 2016; Xu et al. 2016). The increased enrichment of H3K27ac in the *ScF3′H* promoter suggests that the expression of flavonoid metabolism genes is modulated by the level of H3K27ac.

To investigate the regulation of ScWRKY41 on H3K27ac levels in the *ScF3′H* promoter, we performed ChIP-qPCR analysis by overexpressing *ScWRKY41* in hairy root. The results demonstrated that *ScWRKY41* OE significantly enhanced H3K27ac levels in the *ScF3′H* promoter, notably within the region containing the W-box *cis*-elements (Fig. 6F). These results indicate that ScWRKY41 could recruit ScHAC1 to the *ScF3′H* promoter, consequently enhancing H3K27ac levels and thereby stimulating *ScF3′H* expression.

## Discussion

Flavonoids, which are among the most bioactive plant secondary metabolites, play a crucial regulatory role in the ability of plants to adapt to the environment by enhancing antioxidant activity, thereby mitigating or eliminating damage caused by ROS that accumulate as a result of adverse external stimuli (Hernandez et al. 2009; Agati et al. 2011). Flavonoids are ROS scavengers, effectively trapping and neutralizing radicals due to the number and arrangement of their hydroxyl groups on their ring structures (Winkel-Shirley 2002). Our findings indicate that ScF3′H, a key enzyme responsible for the hydroxylation of flavonoids at the 3′ position, plays a pivotal role in modulating antioxidant activity and enhancing freezing tolerance in potato (Fig. 1 and Fig. 2). *ScF3′H* OE altered the flavonoid metabolic pathway, leading to an enhanced conversion of kaempferol-related flavonoids into quercetin-related flavonoids (Fig. 3). Kaempferol and quercetin are typical flavonoids with the same A and C ring structures but different B ring hydroxylation. Quercetin exhibits stronger hydroxyl radical (•OH) scavenging properties than kaempferol, suggesting that radical scavenging activity is enhanced by an increased number of −OH groups on the aromatic B ring (Husain et al. 1987). Therefore, *ScF3′H* affects the downstream metabolism of quercetin and kaempferol to enhance freezing resistance in potato.

In the cultivated variety “Desiree”, flavonoid metabolism exhibited minimal responsiveness to cold, and *ScF3′H* OE did not significantly alter flavonoid metabolic flux under cold conditions (Fig. 3 and Supplementary Table S3). Based on this, we concluded that the cold-induced transcription of *ScF3′H* is a crucial factor contributing to cold tolerance in CM (Fig. 1A). The MYB-bHLH-WD repeat (MBW) transcriptional complex and WRKY transcription factors have been implicated in flavonoid metabolism regulation in potato tubers (Zhang et al. 2021, 2024; Liu et al. 2023a, 2023b), whereas the underlying molecular mechanism governing flavonoid responses to cold stress in potato plants remains unclear. We found that in wild potato CM, cold induced the expression of ScWRKY41, which bound to the *ScF3′H* promoter region that was rich in W-box *cis*-elements, thereby activating *ScF3′H* expression and enhancing cold stress tolerance in potato (Fig. 4 and Fig. 5). WRKY transcription factors have been reported to regulate flavonoid metabolism in potato and other species (Cao et al. 2024). StWRKY13 and StWRKY70 promote anthocyanin accumulation in potato tubers (Zhang et al. 2021, 2024), and MdWRKY11 promotes flavonoid accumulation in apple (Wang et al. 2018). AtWRKY53 binds to the W-box in the *AtDFR* and *AtCHS* promoters to regulate gene expression in Arabidopsis (Chen et al. 2019). VqWRKY31 from *Vitis quinquangularis* increases flavonoid and stilbene accumulation by promoting *VvCHS*, *VvCHI*, *VvDFR*, and *VvFLS* expression, which enhances powdery mildew resistance (Yin et al. 2022). GhWRKY41 forms a homodimer to activate itself and cinnamate 4-hydroxylase (GhC4H) expression, promoting flavonoids and lignin accumulation and improving resistance to *Verticillium dahliae* in cotton (Xiao et al. 2023). These studies collectively imply that WRKYs have a highly conserved function in regulating flavonoid metabolism in plants and play pivotal roles in modulating plant growth, development, and stress tolerance.

**Figure 7.**
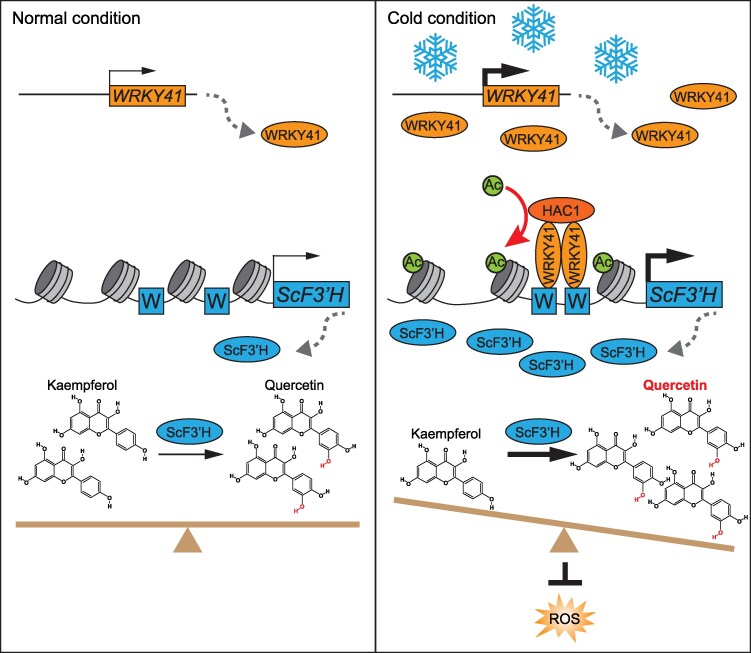
Working model: the ScWRKY41-HAC1 complex activates *ScF3′H* to affect flavonoid metabolism in response to cold stress. After freezing tolerant species *S. commersonii* perceives the cold signal, ScWRKY41 binds to the W-box *cis*-elements and recruits ScHAC1 into the promoter region of *ScF3′H* through physical interactions. Subsequently, ScF3′H boosts the production of quercetin derivatives and enhances the antioxidant capacity, thereby improving cold resistance in potato. Dashed arrows in the figure indicate the process of gene transcription and translation, while sharp arrows indicate promotion; the thickness of the arrows represents the intensity of inhibition or promotion. Blunt arrows (┴) in the figure indicate inhibition.

Our results indicate that the *ScWRKY41* significantly increases after a 3 h treatment at 4 °C compared with the control at 22 °C (Fig. 4E). We hypothesize that the expression of *ScWRKY41*, similar to many key plant stress-responsive genes, is regulated by a negative feedback mechanism in cold response (Hu et al. 2013; Li et al. 2017a, 2017b; Zhang et al. 2022). This mechanism is characterized by an initial transient induction upon the perception of stress signals, followed by a subsequent return to near-basal expression levels. This regulatory likely serves to prevent the prolonged high-level activation of certain stress response pathways, thereby mitigating potential adverse effects. WRKY transcription factors form homo- or hetero-dimers to execute their functions and modulate target gene transcription by recruiting transcription cofactors to histones in promoter regions (Cheng et al. 2021; Mahiwal et al. 2024). AtWRKY38 and AtWRKY62 interact with histone deacetylase 19 (AtHDA19) to coordinately regulate defense responses, and AtWRKY18 and AtWRKY53 form a complex with AtHAC1 to fine-tune plant growth in response to sugar signaling (Kim et al. 2008; Chen et al. 2019). The present study revealed that ScWRKY41 forms homodimers in the nucleus, specifically binding to the W-box-rich region within the *ScF3′H* promoter, subsequently enhancing the H3K27ac modification level of *ScF3′H* by recruiting ScHAC1 under cold conditions, and thereby activating *ScF3′H* expression (Fig. 7). This study addressed a knowledge gap regarding the molecular mechanism underlying the cold response of flavonoid metabolism in wild potato. It provides a foundation for further exploration of wild potato genetic resources aimed at enhancing the environmental adaptability of cultivated potatoes.

## Materials and Methods

### Plant materials and growth conditions

The genotypes used in the present study included *S*. *tuberosum* L. (DM1-3 516R44, DM), *S*. *commersonii* (CIP762471, CIP762476, and CIP761098), and *S*. *tuberosum* L. cv. Desiree (4×). Plants were cultured in vitro for propagation on Murashige and Skoog (MS) medium supplemented with 3% sucrose and 0.31% Gelzan (G3251, PhytoTechnology Laboratories) at 20 ± 1 °C and 60 *μ*mol m^−2^ s^−1^. The 2-wk-old plantlets were transplanted into plastic pots (10 × 10 cm) in a climate room at 22 ± 2 °C under long-day (LD) conditions (16/8-h light/dark).

### Plasmid construction and transformation

Two guide RNAs (gRNAs) for *ScF3′H* were selected from *S*. *commersonii* (CIP762471, CM) and used for BLAST analysis against potato reference genome version 6.1 (Pham et al. 2020). No other potential matches were identified in the genome. The two gRNAs were designed and constructed using a polycistronic tRNA-gRNA (PTG) approach. Specific primer design and the Golden Gate assembly protocol were based on the PTG construction method (Xie et al. 2015). The full-length CDS of *ScF3′H* was cloned from CM and inserted into the pCAMBIA2300 binary vector linearized with *Xba*I and *Bam*HI under the control of the Cauliflower mosaic virus (CaMV) 35S promoter. The recombinant plasmid was sequenced and introduced into *Agrobacterium tumefaciens* strain GV3101. Subsequently, it was transformed into *S*. *commersonii* and *S*. *tuberosum* L. cv. Desiree using *Agrobacterium*-mediated genetic transformation. The kanamycin resistance gene *NPTII* was amplified by PCR with primers *NPTII*-F/R to verify the screening results (Supplementary Table S5). *ScF3′H* expression in the transgenic lines was further assayed using RT-qPCR.

### Determination of freezing tolerance

Six-wk-old plants were then moved to 4 °C for 7 d of pretreatment in a growth chamber (BPC500H, JIUPO, Fujian, China). For freezing resistance phenotype analysis in Desiree, OE lines, and CM KO lines, cold-acclimated plants were kept at −2 °C for 12 h and −4 °C for 6 h. Electrolyte leakage was used to assess the response to freezing treatment (Jia et al. 2016). The relative electrolyte leakage was determined in 3 biological replicates, each with 3 technical replicates.

### Metabolome profiling of potato leaves

For flavonoid analysis in transgenic potatoes, leaves were harvested from 6-wk-old Desiree and *ScF3′H*-OE in the control and after being treated at 4 °C for 7 d. The sampled leaves were freeze-dried using a vacuum freeze-dryer and then crushed with a mixer mill (MM400, Retsch, Germany). The resulting lyophilized powder (100 mg) was dissolved in 1.2 mL of a 70% methanol solution and vortexed for 30 s every 30 min, repeating the process 6 times in total. The sample was then placed in a refrigerator overnight at 4 °C. After centrifugation at 12,000 rpm for 10 min, the extracts were filtered (SCAA-104, 0.22 *μ*m pore size; ANPEL, Shanghai, China) before UPLC-MS/MS analysis. The sample extracts were analyzed using an LC-ESI-MS/MS system (UPLC, Shim-pack UFLC SHIMADZU CBM A system; MS, QTRAP 4500 System). The UPLC conditions were as follows: a Waters ACQUITY UPLC HSS T3 C18 column (1.8 *µ*m, 2.1 × 100 mm), temperature of 40 °C, flow rate of 0.4 mL/min, injection volume of 2 *μ*L, and solvent system of water (0.1% formic acid): acetonitrile (0.1% formic acid). DAMs were identified using partial least squares-discriminant analysis (PLS-DA) with a fold-change threshold of > 2 or < 0.5 and variable influence on projection (VIP) values > 1.0.

### Detection of ROS and total antioxidant activity

Hydrogen peroxide (H_2_O_2_) and superoxide radical (O_2_^−^) were detected using histochemical staining with DAB and NBT chloride, as previously described (Romero-Puertas et al. 2004). For both staining methods, the second leaves of Desiree seedlings were decolorized in 95% ethanol and treated at 80 °C for 30 min until the leaf color was completely removed.

The total antioxidant capacity was subsequently determined using a 2,2′-Azinobis-(3-ethylbenzothiazoline)-6-sulfonic acid (ABTS) Test Kit (S0121, Beyotime, China). The assay was performed according to the manufacturer's instructions.

### Determination of anthocyanin content

Anthocyanins exhibit a red color in acidic solutions, with the intensity of the color being proportional to the concentration of anthocyanins. The absorption peak wavelength of anthocyanin acid solution is 530 nm, and the molar extinction coefficient (ε) is 4.62 × 10^4^.

A 0.1-g potato leaf sample was added to 10 mL of hydrochloric acid–ethanol solution (0.1 mol/L) and placed in a water bath at 60 °C for 30 min. Then, 5 mL of hydrochloric acid–ethanol solution was added successively at 15 min and 1 h and finally brought up to a 25-mL volume. Using 0.1 mol/L hydrochloric acid–ethanol solution as the reference solution, the extraction solution was measured at 530−, 620−, and 650-nm wavelengths using Varioskan LUX (Thermo Fisher Scientific). The anthocyanin content was calculated according to the following formula:


$$OD_{\lambda }\;=\;(OD_{530}-OD_{620})-0.1\;\times \;(OD_{650}-OD_{620})$$

$$\mathrm{Anthocyanin}\;\mathrm{content}\;(\mathrm{nmol}/g)\;=\;OD_{\lambda }/\varepsilon \;\times \;V/m\;\times \;10^{6}$$

### Gene expression analysis with RT-qPCR

First-strand cDNA was synthesized based on the previously mentioned total RNAs using HiScript II reverse transcriptase (Vazyme Biotech, Nanjing, China). For RT-qPCR, the SYBR green method was used with the following cycles: 95 °C for 30 s, followed by 40 cycles of 95 °C for 5 s and 58 °C for 30 s and 72 °C for 30 s. The fluorescence intensity was determined using a CFX Connect Real-Time PCR Detection System (Bio-Rad, Hercules, CA, USA). Endogenous *StEF1A* expression was used to calibrate the expression level of the query genes, as an appropriate reference for cold stress in potato (Nicot et al. 2005). The 2^−ΔΔCt^ method was used to calculate the relative expression levels of the target genes. The primers used for RT-qPCR in this study are listed in Supplementary Table S5.

### Yeast one-hybrid and two-hybrid assays

The *pABAi-ScF3′H* recombinant plasmid was digested with *Bst*BI and then transformed into the Y1H Gold yeast strain. After 3 d of culture at 28 °C, the transformed yeast cells were selected on a SD/−Ura selection plate containing 200 ng/mL AbA. The *pGADT7-ScWRKY6/17/30/41* effector plasmids were co-transformed with the *pABAi-ScF3′H* reporter plasmids into positive yeast colonies. After the second transformation, the yeast suspension was uniformly coated on the selected medium (SD/−Ura/−Leu) containing 200 ng/mL AbA.

To generate the Y2H constructs, the full-length coding sequence of *ScWRKY41* was cloned into both pGBKT7 and pGADT7. Both vectors were linearized with *Eco*RI and *Bam*HI. The full-length N-terminal (1 to 2,103 bp) and C-terminal region (2,622 to 5,058 bp) of *ScHAC1* were cloned into pGBKT7. The Y2H assay was performed according to a procedure previously described (Shi et al. 2017). Yeast cells carrying the empty vector (pGADT7-Rec) were cultured as the negative control in the Y2H assay.

### Transient luciferase expression assays

To investigate cold-responsive promoter activity, the *F3′H* promoter was cloned from both DM and CM, and the *ScF3′H* promoter was truncated into 3 distinct fragments, D1, D2, and D3. These *F3′H* promoter fragments of were inserted into the *Xba*I and *Bam*HI sites of the pCAMBIA2300-LUC vector and subsequently transformed into *Agrobacterium rhizogenes* MSU440. Following a 30-d incubation period, positive hairy root screening was conducted to assess the relative LUC activity at 4 °C for 0, 2, 4, and 6 h.

The *pF3′H-D2:LUC* and *pF3′H-D3:LUC* reporter plasmids were combined with the *35S:ScWRKY41* effector to transform *A*. *tumefaciens* GV3101. The *Agrobacterium* suspension was slowly injected into the leaves of *N*. *benthamiana*. After injection, the recipient *N*. *benthamiana* plants were left in the dark overnight and then grown under LD conditions. LUC fluorescence was measured 48 h after injection using 5200Multi (Tanon) and Varioskan LUX (Thermo Fisher Scientific).

### Subcellular localization of ScWRKY41

The CDS sequence of *ScWRKY41* was inserted into the *Xba*I site of the pCAMBIA2300-35S-eYFP vector and subsequently transformed into *A*. *tumefaciens* GV3101. The empty plasmid was operated in the same way as the negative control. The *Agrobacterium* suspension was slowly injected into the leaves of *N*. *benthamiana*. Subcellular localization was observed 48 h after injection using a fluorescence microscope. The nucleus was labeled with 4′,6-diamidino-2-phenylindole (DAPI), which was excited at 405 nm (0.8% laser power), and detected at 400 to 517 nm. Yellow Fluorescent Protein (YFP) was excited at 488 nm (3.4% laser power), and detected at 490 to 575 nm.

### BiFC assays

The full-length coding regions of *ScWRKY41* and *ScHAC1* were separately recombined into the nYFP and cYFP vectors Both vectors were linearized with *Bam*HI and *Sal*I. The fused nYFP and cYFP vectors were separately transformed into *Agrobacterium* GV3101 and then co-infiltrated into *N*. *benthamiana* leaves. After 2 d of incubation, fluorescence signals of YFP in the transfected epidermal cells were imaged using confocal microscopy. DAPI was excited at 405 nm, and detected at 400 to 517 nm. YFP was excited at 488 nm, and detected at 490 to 575 nm.

### ChIP-qPCR assay

ChIP-qPCR was performed according to a previously described procedure (Saleh et al. 2008). To analyze the binding of ScWRKY41 to the *ScF3′H* promoter, the *35S:ScWRKY41-eYFP* construct was delivered into CM hairy roots via *Agrobacterium rhizogenes* MSU440. Following a 30-d incubation period, positive hairy roots were collected and cross-linked for 15 min with 1% formaldehyde under vacuum. Immunoprecipitation was performed using an anti-GFP monoclonal antibody (Sigma, 11814460001). To assess the H3K27ac enrichment at the *ScF3′H* promoter region, CM seedlings were subjected to cold stress for 3 and 24 h, and the analysis was also extended to hairy roots by overexpressing *ScWRKY41*. An anti-H3K27ac antibody (Abcam, ab4729) was used to perform immunoprecipitation. The P1 to P3 fragments of the *ScF3′H* promoter were amplified in the ChIP-qPCR assays (Supplementary Table S5).

### Accession numbers

Sequence data from this article can be found in the potato genome annotation database (DM8.1; http://www.bioinformaticslab.cn/pubs/dm8/) under the following accession numbers: *StF3′H* (DM8C03G29990), *StCHS* (DM8C05G24190), *StCHI* (DM8C05G22750), *StANS* (DM8C08G26790), *StF3H* (DM8C02G23930), *StF3′5′H* (DM8C11G21030), and *StEF1A* (DM8C06G05660).

The protein sequence accession numbers used to construct the evolutionary tree are as follows: AtF3′H (AT5G07990), BrF3′H (BrO_302 V.10G241800), SlF3′H (Solyc03T002802), ZmF3′Ha (Zm00001d017077), ZmF3′Hb (Zm00001d010521) and OsF3′H (LOC_Os10g17260), AtWRKY53 (AT4G23810), AtWRKY41 (AT4G11070), StWRKY53a (DM8C08G04660), SlWRKY53 (Solyc08T000337), StWRKY41 (DM8C08G29010), SlWRKY41 (Solyc08T002465), and StWRKY53b (DM8C08G04680).

## Supplementary Material

kiaf070_Supplementary_Data

## Data Availability

The transcriptome data from this study can be found at the China National Center for Bioinformation (CNCB; https://www.cncb.ac.cn/) under accession number CRA018559.

## Supplementary data

The following materials are available in the online version of this article.

**Supplementary Figure S1.** Analysis of the F3′H protein sequence.

**Supplementary Figure S2.** Identification of *ScF3′H*-OE transgenic potato lines.

**Supplementary Figure S3.** Flavonoid metabolome data of Desiree and *ScF3′H*-OE lines grown at 22 and 4 °C.

**Supplementary Figure S4.** Protein sequence analysis and subcellular localization of ScWRKY41.

**Supplementary Table S1.** DAMs of flavonoids between Desiree and *ScF3′H*-OE grown at 22 °C.

**Supplementary Table S2.** DAMs of flavonoids between Desiree and *ScF3′H*-OE grown at 4 °C.

**Supplementary Table S3.** Changes in quercetin-*O*-gly, kaempferol-*O*-gly, and anthocyanin levels between Desiree and *ScF3′H*-OE lines.

**Supplementary Table S4.** Seventeen *WRKYs* with upregulated expression in CM were identified from the RNA-seq data.

**Supplementary Table S5.** List of primers used in this study.

## Dive Curated Terms

The following phenotypic, genotypic, and functional terms are of significance to the work described in this paper:

F3H Gramene: AT3G51240
F3H Araport: AT3G51240

WRKY11 Gramene: AT4G31550
WRKY11 Araport: AT4G31550

WRKY17 Gramene: AT2G24570
WRKY17 Araport: AT2G24570

WRKY3 Gramene: AT2G03340
WRKY3 Araport: AT2G03340

WRKY6 Gramene: AT1G62300
WRKY6 Araport: AT1G62300

flavonoids CHEBI: CHEBI:72544

WRKY40 Gramene: AT1G80840
WRKY40 Araport: AT1G80840

WRKY30 Gramene: AT5G24110
WRKY30 Araport: AT5G24110

WRKY41 Gramene: AT4G11070
WRKY41 Araport: AT4G11070

CHS Gramene: AT5G13930
CHS Araport: AT5G13930

HAC1 Gramene: AT1G79000
HAC1 Araport: AT1G79000

FLS Gramene: AT5G08640
FLS Araport: AT5G08640

MEGA CHEBI: CHEBI:6617
