# Supplementary material for: The transcription factor WRKY41–FLAVONOID 3′-HYDROXYLASE module fine-tunes flavonoid metabolism and cold tolerance in potato
Source: Plant Physiol. 2025 Feb 20;197(3):kiaf070. doi: 10.1093/plphys/kiaf070 (PMC11879589; doi:10.1093/plphys/kiaf070)
Supplement: kiaf070_Supplementary_Data [file kiaf070_supplementary_data.zip › Supplementary Figures.pdf]

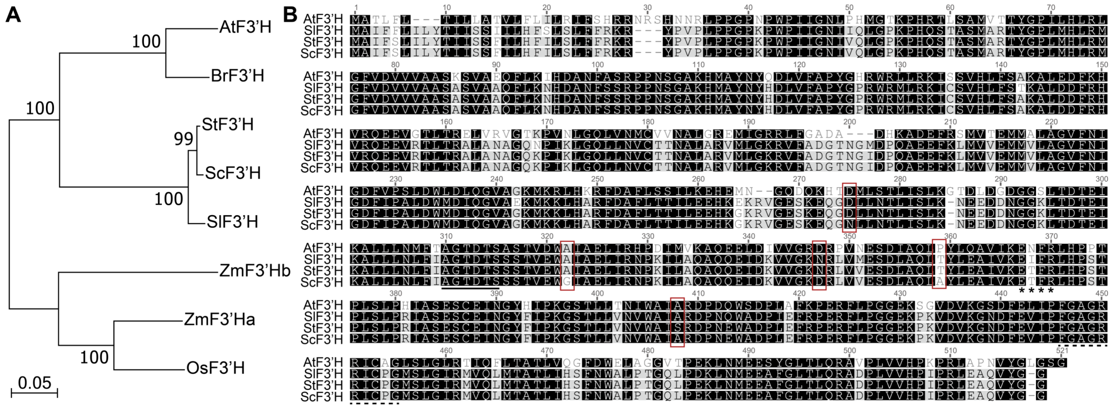

Supplementary Figure S1. Analysis of the F3'H protein sequence.

(A) Phylogenetic tree of F3'H sequences. A neighbor-joining phylogenetic tree was constructed using F3'H protein sequences from various plant species with MEGA 7.0 software. Bootstrap values are based on 1,000 replicates. The F3'H proteins from *Arabidopsis* (AtF3'H), *Solanum lycopersicum* (SlF3'H), *Solanum tuberosum* (StF3'H), and *Solanum commersonii* (ScF3'H), *Brassica rapa* (BrF3'H), *Oryza sativa* (OsF3'H), *Zea mays* (ZmF3'H). The 0.05 scales show substitution distance. **(B)** Sequence alignment of F3'H Proteins. The black line indicates binding pocket motif for oxygen molecules. Dotted line indicates heme-binding domain. The EXXR motif is labeled with an asterisk. Red boxes indicate the amino acid variation of F3'H in DM and CM. Black and grey boxes indicate identical and similar residues, respectively.

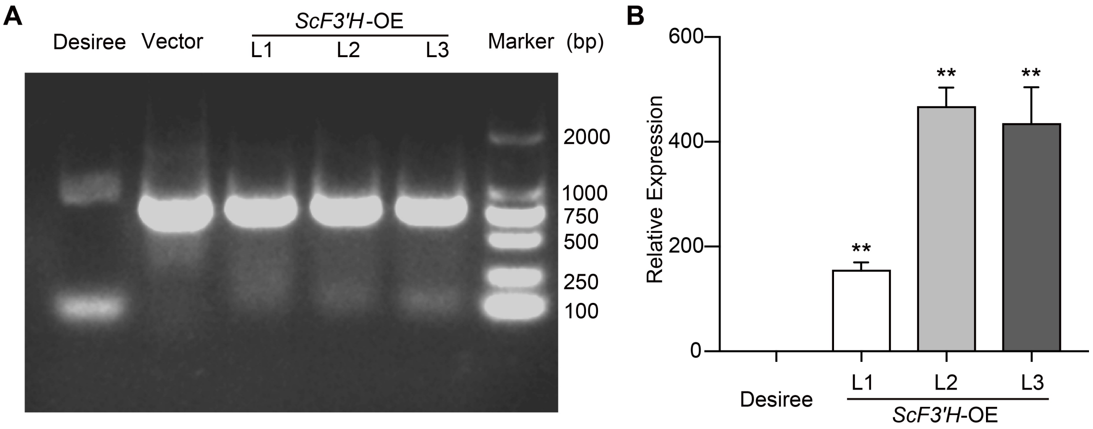

Supplementary Figure S2. Identification of *ScF3'H*-OE transgenic potato lines. **(A)** The kanamycin resistance gene, *NPTII*, was amplified from genomic DNA of Desiree and transgenic plants (lines 1, 2 and 3). The plasmid DNA of vector pCAMBIA2300 was used a positive control. **(B)** The qRT-PCR analysis of *F3'H* expression in *ScF3'H*-OE transgenic *S. tuberosum* 'Desiree'. The data represent the mean ± SE of three independent experiments. The reference gene was *StEF1A*. The asterisk indicates statistically significant differences from control values based on Student's *t* test (**, $P < 0.01$).

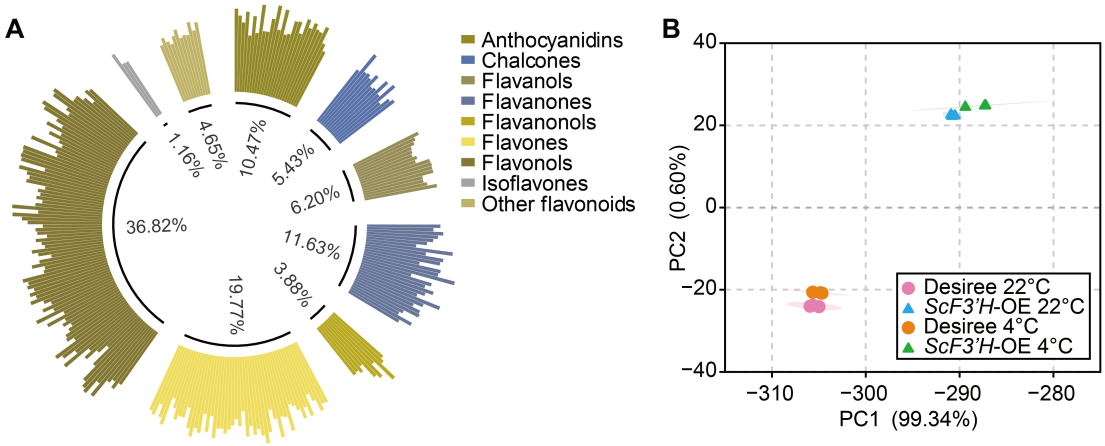

Supplementary Figure S3. Flavonoid metabolome data of Desiree and *ScF3'H*-OE lines grown at 22 and 4°C.

Desiree and *ScF3'H*-OE plants were grown at 22°C for 6 weeks, subjected to 7 days of cold acclimation at 4°C. Samples were collected at 22°C and 4°C for metabolomic analysis. A total of 258 flavonoid metabolites were generated from the Desiree and *ScF3'H*-OE lines. **(A)** The percentage distribution of all flavonoids. **(B)** A principal component analysis (PCA) of all flavonoid metabolites revealed that the accumulation patterns of the Desiree and *ScF3'H*-OE lines exhibited notable distinctions, particularly in the PC1 dimension, which explained 99.34% of the total variation.

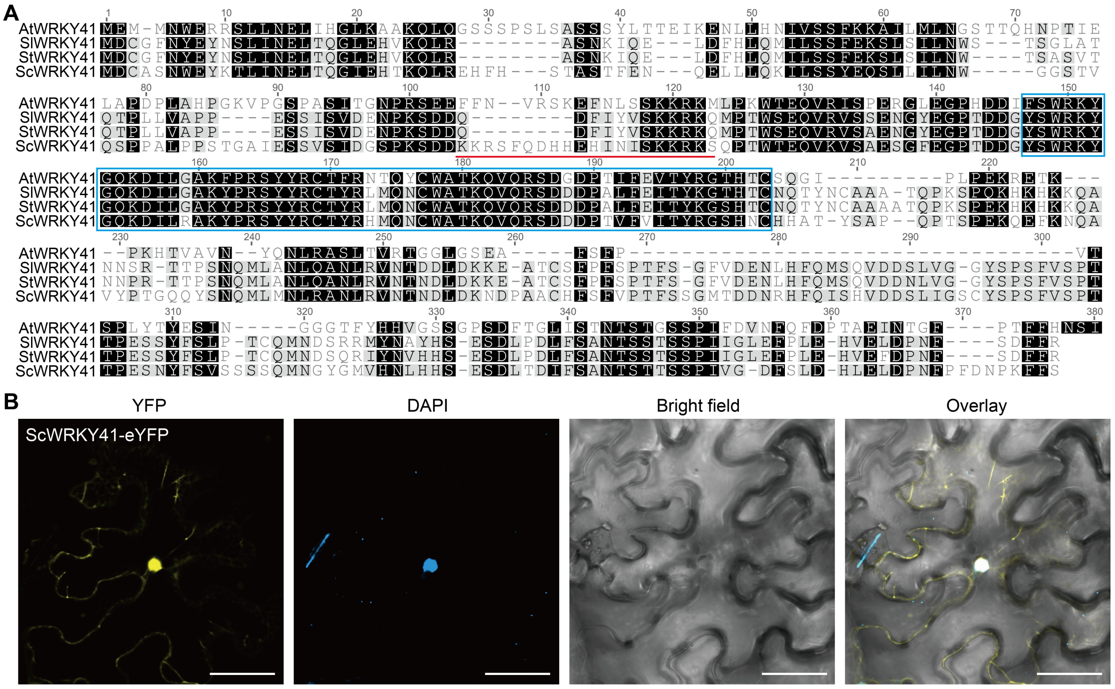

Supplementary Figure S4. Protein sequence analysis and subcellular localization of ScWRKY41.

(A) (A) Alignment of AtWRKY41, SlWRKY41, StWRKY41 and ScWRKY41 from *Arabidopsis*, *S. lycopersicum*, *S. tuberosum*, *S. commersonii*, respectively. The red line indicates the nuclear localization signal peptide. The blue box indicates the conserved domain of the group III WRKY subfamily. Black and grey boxes indicate identical and similar residues, respectively. **(B)** ScWRKY41 is predominantly localized in the nucleus. Tobacco leaves were infected with *A. tumefaciens* carrying the *35S:ScWRKY41-eYFP* construct, and the fluorescent signal of *ScWRKY41-eYFP* in *N. benthamiana* epidermal cell was observed using laser scanning confocal microscopy 48 h post-infection. The nucleus was labeled with 4’,6-diamidino-2-phenylindole (DAPI). The scale bar represents 50 µm.
